# Left Atrial Thrombus and Cardioembolic Stroke in Chagas Cardiomyopathy Presenting with Atrial Flutter: A Case Report

**DOI:** 10.3390/jcm15020456

**Published:** 2026-01-07

**Authors:** Mauricio Sebastián Moreno-Bejarano, Israel Silva-Patiño, Andrea Cristina Aragón-Jácome, Juan Esteban Aguilar, Ana Sofía Cepeda-Zaldumbide, Angela Velez-Reyes, Camila Salazar-Santoliva, Jorge Vasconez-Gonzalez, Juan S. Izquierdo-Condoy, Esteban Ortiz-Prado

**Affiliations:** 1 Centro de Salud, Ministerio de Salud Pública, Lasso 050102, Cotopaxi, Ecuador; 2 Centro de Salud, Ministerio de Salud Pública, Valencia 120610, Los Rios, Ecuador; 3 Centro de Salud, Ministerio de Salud Pública, San Antonio de Pichincha, Quito 170102, Pichincha, Ecuador; 4 One Health Research Group, Universidad de las Américas, Calle de los Colimes y Avenida de los Granados, Quito 170137, Pichincha, Ecuador

**Keywords:** Chagas disease, cardiomyopathy, cardioembolic stroke, atrial thrombus

## Abstract

**Background**: Chagas disease, caused by *Trypanosoma cruzi*, remains endemic throughout Latin America but is increasingly reported in urban areas due to migration and vector adaptation. The cardiac form is the most severe manifestation, associated with arrhythmia, mural thrombus formation, and a high risk of cardioembolic events. Stroke secondary to Chagas cardiomyopathy is uncommon and poses diagnostic and therapeutic challenges. **Case Presentation**: A 58-year-old woman with serologic evidence of *T. cruzi* infection presented with sudden-onset dyspnea, oppressive chest pain, and left-sided weakness. Neurological examination revealed left brachiocrural hemiparesis and mild dysarthria (NIHSS = 9). Non-contrast cranial CT showed an acute infarct in the right middle cerebral artery territory (ASPECTS = 7). Electrocardiography demonstrated typical atrial flutter with variable conduction, and transthoracic echocardiography revealed a markedly dilated left atrium containing a mural thrombus and a left ventricular ejection fraction of 45%. Intravenous thrombolysis with alteplase (0.9 mg/kg) was administered within 4.5 h of symptom onset. Pharmacologic rhythm control was achieved using intravenous and oral amiodarone, followed by oral anticoagulation with warfarin (target INR 2.0–3.0) after excluding hemorrhagic transformation. The patient showed rapid neurological improvement (NIHSS reduction from 9 to 2) and was discharged on day 10 with minimal residual deficit (mRS = 1), sinus rhythm, and stable hemodynamics. **Conclusions**: This case highlights the rare coexistence of Chagas cardiomyopathy, atrial flutter, and cardioembolic stroke due to left atrial thrombus. Early recognition, adherence to evidence-based guidelines, and multidisciplinary management were key to achieving a favorable outcome. Timely diagnosis and intervention remain crucial to preventing severe complications in patients with Chagas disease.

## 1. Introduction

Chagas disease, also known as American trypanosomiasis, is a zoonotic illness caused by the parasite *Trypanosoma cruzi* [1], which enters the human body through direct contact with the feces or urine of its vector, the triatomine insect, commonly referred to as the “kissing bug” [2]. According to data from the World Health Organization (WHO), it is estimated that approximately 7 million people worldwide are infected with this parasite, and more than 100 million individuals are at risk of becoming infected [3]. It is important to note that the vast majority of new infections—likely >90%—remain undiagnosed, and around 70% of infected individuals are unaware of their condition [4].

Currently, two stages of the disease are distinguished: the acute phase and the chronic phase [5]. The acute phase corresponds to the initial period of infection, lasting four to eight weeks. During this stage, most infections are asymptomatic, and only about 10% of patients develop more specific symptoms. Commonly reported symptoms include fever, general malaise, lymphadenopathy, and hepatosplenomegaly [5,6]. Patients who do not receive treatment will progress to the chronic phase of the disease, which can present in four distinct forms: indeterminate, cardiac, digestive, or mixed [1,5,6]. Cardiac myocardiopathy is the most common manifestation of the symptomatic chronic form of the disease [5,7]. Additionally, Chagas disease has been described as an independent risk factor for stroke, regardless of the severity of the cardiomyopathy [8]. Here we present a case of Chagas cardiomyopathy manifesting in the atypical context of atrial flutter and ischemic stroke, highlighting its diagnostic and therapeutic challenges.

## 2. Materials and Methods

This case report was prepared in accordance with the CARE guidelines for case reports to ensure accuracy, transparency, and clinical relevance [9]. Prior to drafting, informed consent was obtained from the patient. The patient was informed that only clinical information would be used in the report and that no identifying data—such as name, home address, phone number, email, or identification number—would be included. Furthermore, it was explained that any images used would be strictly medical in nature, such as electrocardiograms or imaging studies, and that no photographs permitting patient identification would be employed at any time.

## 3. Case Report

A 58-year-old woman with a history of chronic Chagas disease, diagnosed 10 months earlier through positive serology for *Trypanosoma cruzi* (IgG), presented to the emergency department with sudden-onset dyspnea, oppressive chest pain, and abrupt weakness of the left side of the body. She had not received specific etiologic treatment and had irregular outpatient follow-up. Her symptoms began abruptly at rest and were accompanied by rapid, irregular palpitations and a transient loss of consciousness. In the preceding months, she had experienced self-limited episodes of palpitations and mild exertional dyspnea.

She had no significant past medical history, no known allergies, and no diagnosis of structural heart disease. She denied alcohol or illicit drug use and reported no family history of premature cardiovascular disease. On the day of symptom onset, she experienced sudden, intense retrosternal pressure radiating to the cervical region, accompanied by severe dyspnea, mild dizziness, and sudden left-sided weakness. Her relatives noticed deviation of the lip commissure and difficulty articulating speech, prompting immediate transfer to the emergency department.

On physical examination, her Glasgow Coma Scale score was 11/15 (E: 4, V: 3, M: 4). She exhibited marked dyspnea, mild dysarthria, and left-sided hemiparesis predominantly affecting the arm and leg, with a Daniels scale score of 3/5. Vital signs showed a blood pressure of 150/90 mmHg, heart rate of 120 bpm, respiratory rate of 24 breaths per minute, oxygen saturation of 89% on room air, and an axillary temperature of 36.8 °C. Skin perfusion was normal. Neurological examination revealed leftward deviation of the lip commissure and hyperreflexia. Cardiac auscultation demonstrated a rapid regular rhythm without murmurs. Peripheral pulses were symmetric, and lung examination showed preserved vesicular breath sounds without added noises. The remainder of the physical examination was unremarkable.

### 3.1. Diagnostic Approach

Upon arrival at the emergency department, a stroke code was activated. The initial National Institutes of Health Stroke Scale (NIHSS) score was 9, determined by left brachiocrural hemiparesis (3 points), facial deviation (2 points), mild dysarthria (1 point), and mild weakness of the left leg (3 points). The Glasgow Coma Scale (GCS) score was 11/15 (E4, V3, M5), and the pre-event modified Rankin Scale (mRS) score was 0, indicating complete baseline functional independence (Table 1).

Following the recommendations of the American Heart Association/American Stroke Association (AHA/ASA), a 12-lead electrocardiogram was performed within the first 10 min, and a non-contrast head computed tomography scan was obtained within the first 25 min after arrival.

**Figure 1:**
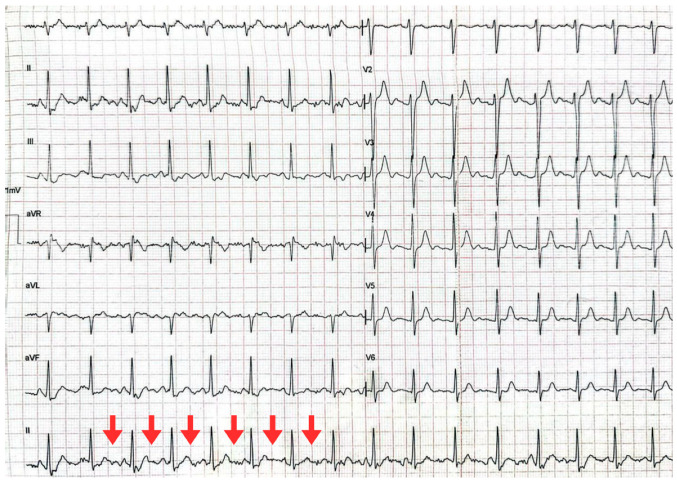
Twelve-lead electrocardiogram obtained at admission. The electrocardiogram shows absence of P waves, a regular rhythm, and a sawtooth pattern (red arrows), consistent with atrial flutter with variable conduction.

The 12-lead electrocardiogram showed a regular non-sinus rhythm, with a ventricular rate of 110 bpm, narrow QRS complexes, absence of P waves, and a sawtooth pattern, most evident in leads II, III, and aVF. These findings were consistent with typical atrial flutter with variable conduction and rapid ventricular response (Figure 1). In interpreting the tracing, atrial fibrillation with regular conduction and atrial tachycardia were considered as differential arrhythmic diagnoses, while non-arrhythmic factors, such as motion artifact or muscle tremor, were recognized as possible sources of confounding during ECG acquisition, but not as alternative diagnoses.

**Figure 2:**
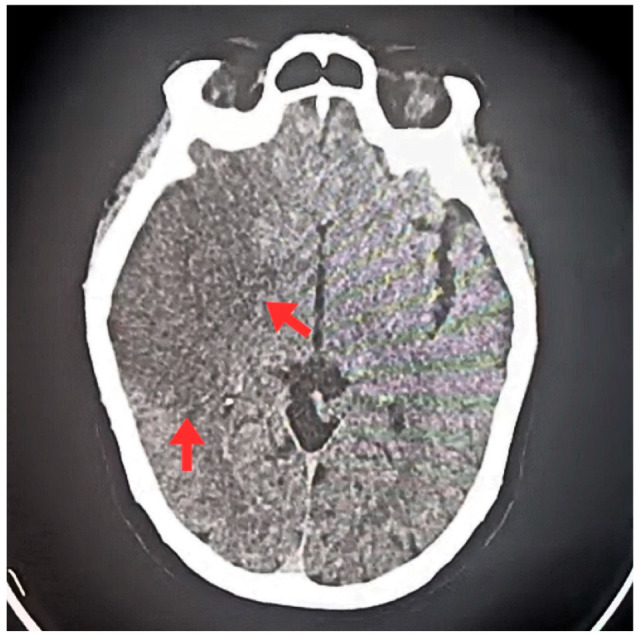
Non-contrast computed tomography of the brain at admission. The scan shows findings compatible with an ischemic stroke in the right frontoparietal region (red arrows), correlating with the patient’s predominantly left-sided neurological symptoms and consistent with contralateral lateralization of the motor deficit.

Non-contrast brain computed tomography revealed cortical and subcortical hypodensity in the territory of the right middle cerebral artery, with hypodense areas in the insular region, lentiform nucleus, head of the caudate nucleus, and adjacent frontoparietal region. These findings were accompanied by cortical sulcal effacement and loss of gray-white matter differentiation. No signs of intracranial hemorrhage, mass effect, or midline shift were observed, consistent with an ischemic stroke (Figure 2). The Alberta Stroke Program Early CT Score (ASPECTS) was 7, indicating moderate acute ischemia in the M1–M3 segments of the right middle cerebral artery territory.

**Figure 3:**
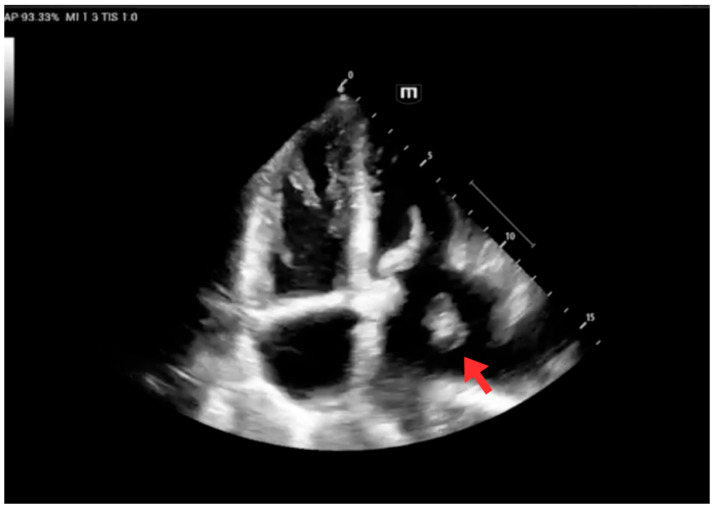
Transthoracic echocardiogram at admission. The study shows cardiac chamber dilation and a mobile thrombus in the left atrium (red arrow).

Transthoracic echocardiography revealed severe left atrial dilation with a mobile thrombus within the chamber. The left ventricular ejection fraction (LVEF) was 45%, with no evidence of ventricular thrombi or apical aneurysm (Figure 3).

Initial laboratory tests showed hemoglobin of 13.4 g/dL, leukocytes 8200/μL, platelets 235,000/μL, capillary glucose 108 mg/dL, and serum creatinine 0.72 mg/dL (CKD-EPI estimated glomerular filtration rate 92 mL/min/1.73 m^2^). Hematologic and coagulation indices were within normal ranges, with no evidence of active infection or acute metabolic disturbances. A mild elevation of troponin I (0.12 ng/mL; reference value < 0.03 ng/mL) was observed, likely associated with myocardial injury secondary to left atrial overload and hemodynamic stress from the embolic event. Elevated NT-proBNP (920 pg/mL) supported the presence of diastolic dysfunction and left atrial dilation observed on echocardiography. Serum electrolytes and renal function were normal, allowing safe use of thrombolytics and antiarrhythmics. Inflammatory markers were slightly elevated, consistent with an acute non-infectious event.

**Figure 4:**
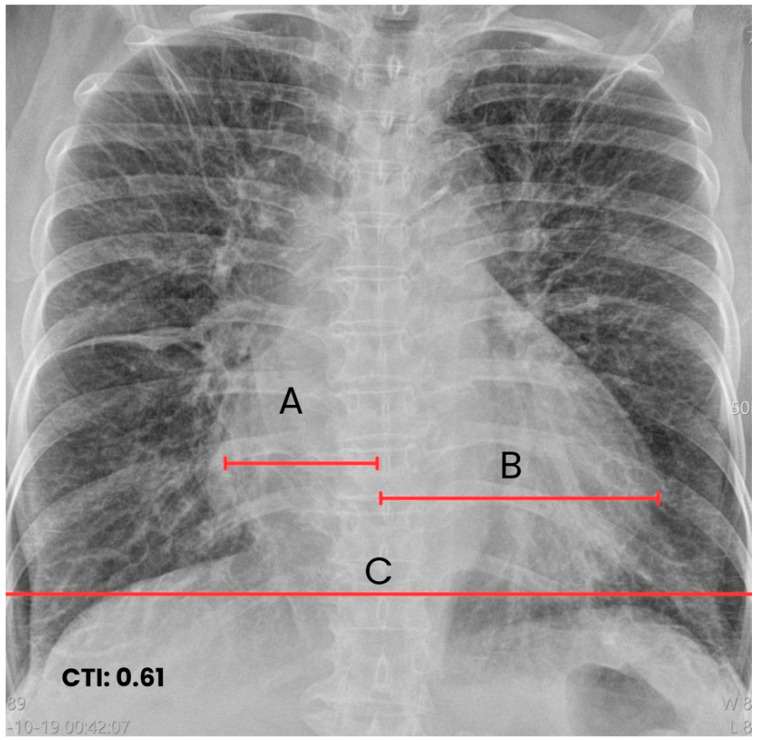
Posteroanterior chest radiograph. The image shows mild cardiomegaly with a cardiothoracic ratio of 0.61 (A + B/C), compatible with grade III cardiomegaly (red lines).

Considering the history of positive *Trypanosoma cruzi* serology (IgG) and the suspicion of chronic Chagas cardiomyopathy, a posteroanterior chest radiograph was obtained. The study showed mild cardiomegaly, with a cardiothoracic ratio of 0.61, compatible with global cardiac chamber dilation. Notably, the presence of the right double contour sign was observed, a classic finding suggesting left atrial enlargement due to superimposition of its medial borders. Pulmonary parenchyma showed no signs of congestion, vascular redistribution, or Kerley lines, indicating a compensated phase of Chagas cardiomyopathy, without radiological evidence of overt heart failure (Figure 4).

Attributing a cardioembolic origin posed a diagnostic challenge due to the coexistence of multiple thrombogenic substrates. Nevertheless, persistent electrocardiographic documentation of atrial flutter—characterized by organized atrial activity, regular RR intervals, and the absence of an irregularly irregular rhythm—allowed atrial fibrillation to be excluded during clinical evaluation. In the setting of Chagas cardiomyopathy, where atrial flutter is typically sustained by macroreentrant circuits overlying atrial fibrosis, this arrhythmia assumes emboligenic significance by virtue of mechanically ineffective atrial contraction and resultant blood stasis [10]. The systematic exclusion of alternative embolic sources—including left ventricular apical aneurysm and paradoxical embolism—supported attribution of the event to atrial flutter as the primary mechanism. Accordingly, the ischemic stroke was classified as cardioembolic according to the TOAST criteria, supported by the coexistence of atrial flutter and Chagas cardiomyopathy, both recognized as major sources of cardiac embolism [11].

### 3.2. Therapeutic Intervention

Once the diagnosis of cardioembolic ischemic stroke secondary to a left atrial thrombus in the context of Chagas cardiomyopathy with atrial flutter was confirmed, immediate treatment was initiated following international recommendations for acute stroke [12]. The patient was transferred to the intensive care unit for close neurological and cardiological monitoring.

Given that the onset of symptoms was less than 4.5 h prior, there were no formal contraindications, and initial neuroimaging ruled out intracranial hemorrhage, intravenous thrombolysis with alteplase (0.9 mg/kg; 10% bolus followed by a 90% infusion over 60 min) was administered, in accordance with the American Heart Association/American Stroke Association (AHA/ASA) guidelines for the management of acute ischemic stroke [12]. While thrombolysis in the presence of an intracavitary thrombus is a controversial decision and not part of standard management, it should be individualized through a risk-benefit analysis, particularly in cases of clinically relevant neurological deficits and eligibility for reperfusion [13].

In Chagas cardiomyopathy, this analysis is especially complex due to the marked thrombogenic substrate, the frequent coexistence of atrial arrhythmias, and the risk of persistent embolization under conservative management. Although specific evidence is limited and restricted to small series and case reports, the available data suggest favorable functional outcomes without a significant increase in bleeding risk [14,15]. Furthermore, there is no data to support the routine use of mechanical thrombectomy in acute ischemic stroke associated with Chagas cardiomyopathy, which prevents establishing an evidence-based preference over intravenous thrombolysis. In this context, conservative management without reperfusion is reserved for patients with absolute contraindications, accepting a higher risk of neurological progression or embolic recurrence [15,16,17,18]. During the infusion, the patient remained hemodynamically stable, without neurological deterioration or adverse events.

Simultaneously, due to atrial flutter with rapid ventricular response, pharmacological management of rate and rhythm was initiated with intravenous amiodarone, chosen for its efficacy in controlling atrial and ventricular arrhythmias in Chagas cardiomyopathy [19,20]. An initial bolus of 150 mg over 10 min was administered, followed by a continuous infusion of 1 mg/min for 6 h and then 0.5 mg/min for 18 h, according to American College of Cardiology/American Heart Association (ACC/AHA) recommendations for supraventricular arrhythmia management [21]. This approach achieved a progressive reduction in heart rate and improved clinical tolerance. Continuous cardiac monitoring and strict observation of vital signs and neurological parameters were maintained.

After the acute phase and confirmation of absence of hemorrhage on 24 h follow-up CT, anticoagulation was considered based on thromboembolic and bleeding risk profiles. The patient’s CHA_2_DS_2_-VASc score (3 points) supported permanent anticoagulation, while the HAS-BLED score (1 point) indicated low bleeding risk. Warfarin was initiated with gradual titration to achieve a therapeutic INR of 2.0–3.0 under close monitoring. Warfarin has demonstrated efficacy in reducing recurrent embolic events in this context, and its use is supported by guidelines, particularly in the presence of documented intracardiac thrombus or history of cardioembolic stroke [15,18,22].

Oral amiodarone was continued as maintenance rhythm therapy. The patient progressed without hemorrhagic complications, with sustained hemodynamic stability and gradual neurological improvement. No recurrent embolic events occurred during hospitalization.

### 3.3. Clinical Follow-Up and Outcomes

During her stay in the intensive care unit, the patient remained hemodynamically stable under continuous electrocardiographic and neurological monitoring, with strict control of blood pressure, glucose, temperature, and oxygen saturation. In the first 24 h after thrombolysis, no neurological deterioration or clinical signs of intracranial hemorrhage were observed, and follow-up CT confirmed stability of the ischemic infarct without hemorrhagic transformation.

At 48 h, significant neurological improvement was noted, with increased strength in the left side of the body (Daniels scale 4+/5) and complete resolution of dysarthria. The NIH Stroke Scale (NIHSS) score decreased from 9 to 3 points, while the Glasgow Coma Scale (GCS) remained 15/15 throughout hospitalization, indicating neurological stability and full consciousness.

Follow-up transthoracic echocardiography revealed the persistence of a stable, partially organized left atrial thrombus, smaller than in previous studies, with well-defined borders, homogeneous echogenicity, and no significant mobility, without findings suggestive of fragmentation. These changes are consistent with a process of progressive organization under anticoagulation and suggest a favorable evolution, with a decrease in embolic risk. The LVEF remained at 48%, with no deterioration of ventricular function or the appearance of new structural abnormalities; therefore, continued serial echocardiographic follow-up was recommended. In this context, treatment with oral anticoagulation with warfarin was continued, adjusted to maintain a target INR of 2.0–3.0, with a minimum planned duration of 3 to 6 months, contingent upon complete resolution of the left atrial thrombus documented by echocardiography. Given the presence of atrial arrhythmia and the associated thromboembolic risk, the need for long-term anticoagulation will be reassessed after clinical and imaging follow-up.

By day seven, the patient achieved a modified Rankin Scale (mRS) score of 1, reflecting minimal disability without relevant functional limitation. She was discharged on hospital day ten with NIHSS 2 and mRS 1, in stable clinical condition, without limiting motor deficits or incapacitating residual symptoms. Outpatient follow-up was arranged with neurology, cardiology, and physical rehabilitation services, including serial INR monitoring and planned echocardiographic evaluation.

As shown in Table 2, the patient demonstrated favorable clinical evolution, with sustained neurological recovery, cardiovascular stability, and absence of hemorrhagic or embolic complications. Comprehensive management, including intravenous thrombolysis, rhythm control with amiodarone, and oral anticoagulation, resulted in an optimal functional outcome at hospital discharge, highlighting the effectiveness of a multidisciplinary approach in cardioembolic stroke secondary to Chagas cardiomyopathy, even in the presence of a left atrial thrombus.

## 4. Discussion

The main complications occur during the chronic phase of the disease, such as disability resulting from chronic cardiomyopathy and stroke [18]. In Ecuador, there is limited information available regarding the chronic phase of Chagas disease, as well as studies on Chagas cardiomyopathy. A 2023 study analyzing data from 2011 to 2021 reported that 118 patients with Chagas disease were hospitalized in Ecuador. The study also reported an in-hospital mortality rate of 69.4% (n = 82) [2].

The cardiac form is the most severe and frequent manifestation of chronic Chagas disease; it develops in 20–30% of infected individuals within 10 to 30 years after infection. It typically causes conduction system abnormalities, bradyarrhythmias and tachyarrhythmias, apical aneurysms, heart failure, thromboembolism, and sudden death [23]. Regarding strokes, in a study including 5447 autopsies, 524 corresponded to patients with heart failure secondary to Chagas disease, in whom cerebral infarction was identified in 17.5% of cases [24]. In another study involving 565 patients, the incidence of stroke was higher in patients with Chagas disease compared to those without the disease, with 20.2 versus 13.9 events per 1000 patient-years, respectively [25].

Cardo-Aratal and Gascon describe the main risk factors for the development of cardioembolic stroke in Chagas disease, which include cardiomyopathy (left atrial dilatation; progressive heart failure—left ventricular systolic or diastolic dysfunction; segmental lesions such as left ventricular posterior wall lesions and apical aneurysm); arrhythmias (right bundle branch block often associated with left anterior hemiblock, advanced atrioventricular block, atrial fibrillation, and sustained ventricular tachycardia); and mural thrombus [18]. Oliveira-Filho, in turn, notes that existing theories fail to fully explain the specific nature of cerebral involvement observed in patients with Chagas disease and proposes two mechanisms. The first involves structural damage to the heart caused by *Trypanosoma cruzi*, leading to intracardiac thrombosis and subsequent cerebral embolization. The second involves chronic inflammation, acting mainly through Th1-type cytokines, which may accelerate atherosclerosis and ultimately lead to ischemic stroke [26].

On the other hand, it has been described that excessive nitric oxide release due to increased inducible nitric oxide synthase (iNOS) activity may suppress endothelial nitric oxide synthase activity, resulting in vasoconstriction, cerebral microvascular spasms, and even ischemic stroke [27]. Furthermore, reduced expression of GATA-3, FoxP3, and IL-10, together with increased mRNA expression of IFN-γ, TNF-α, iNOS, has been associated with a higher risk of stroke-related mortality in patients with chronic Chagas disease [28].

Atrial flutter is less frequent than atrial fibrillation in patients. In a study spanning a 10-year period, 2.4% of patients with Chagas disease presented with atrial flutter, while 31.7% had atrial fibrillation with a rapid ventricular response, and 22.4% had atrial fibrillation with a normal ventricular response [29]. However, it has been reported that up to 40% of the general population with atrial flutter eventually develop atrial fibrillation [30]. Additionally, pharmacologic rate control is more difficult to achieve in atrial flutter than in atrial fibrillation [31]. It is important to note that no published cases have been identified of patients with Chagas disease who developed a cardioembolic stroke and, at the same time, presented with atrial flutter.

Current guidelines recommend initiating specific antiparasitic therapy for all cases only if patients present with the indeterminate chronic form (ICF) of Chagas disease. This is because the prognosis of these patients is similar to that of the general population [32]. The recommended regimen includes oral benznidazole 5 mg/kg/day in 2–3 doses for 60 days or oral nifurtimox 10 mg/kg/day in 3 doses for 90 days [33,34]. This practice is justified because the decline in serum antibody titers occurs slowly, and possible reinfections in endemic areas, along with the lack of reliable clinical markers, have limited our understanding of the true efficacy of treatment [35].

However, based on the organic progression observed in the patient in this case, it is preferable to avoid prescribing antitrypanosomal therapy, as the risk of drug toxicity outweighs the potential benefits [32]. Available evidence suggests that in patients with heart failure (HF) stage C or D (according to AHA/ACC staging), prescribing therapy with benznidazole or nifurtimox is not recommended, due to the lack of clear therapeutic benefit. In comparison, patients in stage B AHA/ACC or those with less severe chronic Chagas disease (Rassi score < 7) may be considered for initiating therapy [23,32].

On the other hand, in a study in which a Cox regression analysis was performed to create a risk score related to the annual incidence of cardioembolic ischemic stroke, the risk–benefit analysis recommended prophylaxis with warfarin for cardioembolic stroke in Chagas disease patients with a score of 4 to 5 points [36]. Another study revealed that patients treated with warfarin have a higher proportion of international normalized ratios (INR) within the therapeutic range [37].

Diagnosing Chagas disease in patients presenting with stroke, particularly those from endemic areas or with relevant travel history, is essential [16]. Additionally, educational programs targeting both the general public and healthcare providers are necessary regarding this complication in patients infected with *T. cruzi*, as awareness of stroke risk in individuals with Chagas disease is estimated to be less than 5% [38].

## 5. Conclusions

This case represents the natural progression of untreated Chagas disease, in which the absence of timely intervention led to one of its most severe complications: cardioembolic stroke. The coexistence of Chagas cardiomyopathy, atrial flutter, and left atrial thrombus reflects the advanced spectrum of cardiac involvement in this infection. This clinical scenario, associated with high morbidity and mortality, underscores the need for a high index of diagnostic suspicion—particularly in endemic regions—and for timely complementary studies to identify and manage thromboembolic manifestations before irreversible neurological damage occurs.

The patient’s hemodynamic and neurological stabilization was achieved through strict adherence to evidence-based international guidelines, including intravenous thrombolysis, rhythm control with amiodarone, and anticoagulation with warfarin. This multidisciplinary approach enabled full functional recovery without hemorrhagic complications or recurrent embolic events. Therefore, this case reinforces that early detection, comprehensive management, and adherence to therapeutic protocols are essential pillars for altering the clinical course of Chagas disease and reducing the impact of its cardiovascular and neurological complications in vulnerable populations. Finally, it is important to note that, since this involved a single case and lacked a control group, these results cannot be generalized.

## Figures and Tables

**Table 1:** Initial neurological assessment using standardized scales in acute stroke.

Scale	Assessed Parameters/Relevant Subcomponents	Score Obtained	Total Possible Score	Clinical Interpretation
National Institutes of Health Stroke Scale (NIHSS)	Level of consciousness (1a–1c): 0	9	0–42	Moderate neurological deficit
	Eye movements and visual fields (2–3): 0			
	Facial palsy (4): 2 (left deviation)			
	left arm motor function (5a): 3			
	left leg motor function (6a): 3			
	Language (9): 0 (no aphasia)			
	Dysarthria (10): 1 (mild)			
	Other items (ataxia, sensation, extinction): 0			
Glasgow Coma Scale (GCS)	Eye opening (O): 4	11/15	3–15	Preserved consciousness; patient alert and oriented
	Verbal response (V): 5			
	Motor response (M): 6			
Modified Rankin Scale (mRS)	0: No symptoms or functional limitations	0	0–6	Complete baseline functional independence before the event
	1: Mild symptoms without significant disability			
	2–5: Mild to severe disability			

NIHSS: quantifies the severity of neurological deficit in acute stroke (mild: 1–4; moderate: 5–15; severe: >16). GCS: assesses the level of consciousness (mild: 13–15; moderate: 9–12; severe: ≤8). mRS: measures functional disability (0 = no symptoms; 6 = death).

**Table 2:** Clinical and Therapeutic Summary of Patient Evolution from Admission to Discharge.

Time Point	Setting	Key Clinical Events & Findings	Diagnostics	Treatment	Outcome/Trajectory
−10 months	Outpatient	Chronic Chagas disease confirmed (*T. cruzi* IgG+). No etiologic treatment; irregular follow-up.	Serology	None (etiologic)	—
Months pre-admission	Outpatient	Recurrent, self-limited palpitations and mild exertional dyspnea. No known structural heart disease.	—	—	—
T0 (symptom onset, at rest)	Community	Sudden oppressive chest pain, severe dyspnea, rapid irregular palpitations, transient loss of consciousness; acute left-sided weakness with dysarthria and facial deviation.	—	—	—
T0 + ED arrival	Emergency Dept.	Stroke code activated. NIHSS 9, GCS 11/15, premorbid mRS 0.	ECG: typical atrial flutter with rapid ventricular response. Non-contrast CT brain: right MCA ischemic stroke (ASPECTS 7), no hemorrhage.	Supportive acute stroke care	Acute ischemic stroke suspected/confirmed
Early inpatient workup (Day 0)	Hospital	Suspected cardioembolic source in context of arrhythmia/Chagas cardiomyopathy.	TTE: severe LA dilation + mobile LA thrombus; LVEF 45%. Labs: no major abnormalities; mild troponin I and NT-proBNP elevation.	—	Cardioembolic mechanism supported
≤4.5 h from T0	Hospital	Cardioembolic ischemic stroke diagnosis.	—	IV alteplase (thrombolysis). IV amiodarone for rhythm/rate control.	Stabilization after reperfusion therapy
First 24 h	Hospital	Hemodynamic and neurological stability.	Follow-up CT: no hemorrhagic transformation.	Continue monitoring/management	No bleeding complications documented
48 h	Hospital	Marked neurological improvement.	—	—	NIHSS 3; dysarthria resolved; left strength improved (Daniels 4+/5)
Hospital course (after hemorrhage exclusion)	Hospital	Secondary prevention initiated.	Risk scores documented: CHA_2_DS_2_-VASc 3, HAS-BLED 1.	Warfarin anticoagulation started; transition to oral amiodarone.	Ongoing stability
Follow-up echocardiography	Hospital	Persistent LA thrombus, stable/partially organized; no fragmentation signs.	TTE: persistent thrombus; LVEF 48%.	Continue anticoagulation + rhythm control	Thrombus stable; preserved LV function
Day 7 (discharge)	Discharge	Clinically stable, minimal deficits.	—	Discharged on oral anticoagulation + rhythm control; outpatient follow-up scheduled.	NIHSS 2, mRS 1

Abbreviations: ASPECTS, Alberta Stroke Program Early CT Score; CHA_2_DS_2_-VASc, Congestive heart failure, Hypertension, Age ≥75 years (2 points), Diabetes mellitus, prior Stroke/TIA/thromboembolism (2 points), Vascular disease, Age 65–74 years, Sex category (female); CT, computed tomography; ECG, electrocardiogram; ED, emergency department; GCS, Glasgow Coma Scale; HAS-BLED, Hypertension, Abnormal renal/liver function, Stroke, Bleeding history/predisposition, Labile INR, Elderly (>65 years), Drugs/alcohol; IgG, immunoglobulin G; INR, international normalized ratio; LA, left atrium/left atrial; LVEF, left ventricular ejection fraction; MCA, middle cerebral artery; mRS, modified Rankin Scale; NIHSS, National Institutes of Health Stroke Scale; NT-proBNP, N-terminal pro–B-type natriuretic peptide; *T. cruzi*, *Trypanosoma cruzi*; TIA, transient ischemic attack; TTE, transthoracic echocardiography; IV, intravenous.

## Data Availability

The original contributions presented in this study are included in the article. Further inquiries can be directed to the corresponding author.

## Abbreviations

WHO	World Health Organization
CGS	Glasgow Coma Scale
mRS	modified Rankin Scale
AHA/ASA	American Heart Association/American Stroke Association
ASPECTS	Alberta Stroke Program Early CT Score
NIHSS	National Institutes of Health Stroke Scale
LVEF	left ventricular ejection fraction
ACM	Middle Cerebral Artery
CT	Computed Tomography
ECG	Electrocardiogram
INR	International Normalized Ratio

## References

[1] Winters R., Nguyen T., Waseem M. (2025). Chagas Disease. StatPearls.

[2] Vásconez-González J., Izquierdo-Condoy J.S., Fernandez-Naranjo R., Gamez-Rivera E., Tello-De-la-Torre A., Guerrero-Castillo G.S., Ruiz-Sosa C., Ortiz-Prado E. (2023). Severe Chagas Disease in Ecuador: A Countrywide Geodemographic Epidemiological Analysis from 2011 to 2021. Front. Public Health.

[3] Organizacion Mundial de la Salud Enfermedad de Chagas (Tripanosomiasis Americana). https://www.who.int/es/news-room/fact-sheets/detail/chagas-disease-(american-trypanosomiasis).

[4] Cucunubá Z.M., Gutiérrez-Romero S.A., Ramírez J.-D., Velásquez-Ortiz N., Ceccarelli S., Parra-Henao G., Henao-Martínez A.F., Rabinovich J., Basáñez M.-G., Nouvellet P. (2024). The Epidemiology of Chagas Disease in the Americas. Lancet Reg. Health Am..

[5] Suárez C., Nolder D., García-Mingo A., Moore D.A.J., Chiodini P.L. (2022). Diagnosis and Clinical Management of Chagas Disease: An Increasing Challenge in Non-Endemic Areas. Res. Rep. Trop. Med..

[6] Swett M.C., Rayes D.L., Campos S.V., Kumar R.N. (2024). Chagas Disease: Epidemiology, Diagnosis, and Treatment. Curr. Cardiol. Rep..

[7] Izquierdo-Condoy J.S., Vásconez-Gonzáles J., Morales-Lapo E., Tello-De-la-Torre A., Naranjo-Lara P., Fernández R., Hidalgo M.R., Escobar A., Yépez V.H., Díaz A.M. (2024). Beyond the Acute Phase: A Comprehensive Literature Review of Long-Term Sequelae Resulting from Infectious Diseases. Front. Cell. Infect. Microbiol..

[8] Lage T.A.R., Tupinambás J.T., de Pádua L.B., Ferreira M.d.O., Ferreira A.C., Teixeira A.L., Nunes M.C.P. (2022). Stroke in Chagas Disease: From Pathophysiology to Clinical Practice. Rev. Soc. Bras. Med. Trop..

[9] CARE CARE Case Report Guidelines. https://www.care-statement.org.

[10] Rojas L.Z., Glisic M., Pletsch-Borba L., Echeverría L.E., Bramer W.M., Bano A., Stringa N., Zaciragic A., Kraja B., Asllanaj E. (2018). Electrocardiographic Abnormalities in Chagas Disease in the General Population: A Systematic Review and Meta-Analysis. PLoS Neglected Trop. Dis..

[11] Rathburn C.M., Mun K.T., Sharma L.K., Saver J.L. (2024). TOAST Stroke Subtype Classification in Clinical Practice: Implications for the Get with The Guidelines-Stroke Nationwide Registry. Front. Neurol..

[12] Sacco R.L., Adams R., Albers G., Alberts M.J., Benavente O., Furie K., Goldstein L.B., Gorelick P., Halperin J., Harbaugh R. (2006). Guidelines for Prevention of Stroke in Patients with Ischemic Stroke or Transient Ischemic Attack: A Statement for Healthcare Professionals from the American Heart Association/American Stroke Association Council on Stroke: Co-Sponsored by the Council on Cardiovascular Radiology and Intervention: The American Academy of Neurology Affirms the Value of This Guideline. Stroke.

[13] Powers W.J., Rabinstein A.A., Ackerson T., Adeoye O.M., Bambakidis N.C., Becker K., Biller J., Brown M., Demaerschalk B.M., Hoh B. (2018). 2018 Guidelines for the Early Management of Patients with Acute Ischemic Stroke: A Guideline for Healthcare Professionals From the American Heart Association/American Stroke Association. Stroke.

[14] Nunes M.C.P., Kreuser L.J., Ribeiro A.L., Sousa G.R., Costa H.S., Botoni F.A., Souza A.C.d., Marques V.E.G., Fernandez A.B., Teixeira A.L. (2015). Prevalence and Risk Factors of Embolic Cerebrovascular Events Associated with Chagas Heart Disease. Glob. Heart.

[15] Nunes M.C.P., Beaton A., Acquatella H., Bern C., Bolger A.F., Echeverría L.E., Dutra W.O., Gascon J., Morillo C.A., Oliveira-Filho J. (2018). Chagas Cardiomyopathy: An Update of Current Clinical Knowledge and Management: A Scientific Statement From the American Heart Association. Circulation.

[16] Vásconez-González J., Miño C., Salazar-Santoliva C., Villavicencio-Gomezjurado M., Ortiz-Prado E. (2024). Chagas Disease as an Underrecognized Cause of Stroke: Implications for Public Health. Front. Med..

[17] Trabuco C.C., Pereira de Jesus P.A., Bacellar A.S., Oliveira-Filho J. (2005). Successful Thrombolysis in Cardioembolic Stroke from Chagas Disease. Neurology.

[18] Carod-Artal F.J., Gascon J. (2010). Chagas Disease and Stroke. Lancet Neurol..

[19] Martinelli-Filho M., Marin-Neto J.A., Scanavacca M.I., de Paola A.A.V., Medeiros P.d.T.J., Owen R., Pocock S.J., de Siqueira S.F., CHAGASICS investigators (2024). Amiodarone or Implantable Cardioverter-Defibrillator in Chagas Cardiomyopathy: The CHAGASICS Randomized Clinical Trial. JAMA Cardiol..

[20] Stein C., Migliavaca C.B., Colpani V., da Rosa P.R., Sganzerla D., Giordani N.E., Miguel S.R.P.d.S., Cruz L.N., Polanczyk C.A., Ribeiro A.L.P. (2018). Amiodarone for Arrhythmia in Patients with Chagas Disease: A Systematic Review and Individual Patient Data Meta-Analysis. PLoS Neglected Trop. Dis..

[21] Al-Khatib S.M., Stevenson W.G., Ackerman M.J., Bryant W.J., Callans D.J., Curtis A.B., Deal B.J., Dickfeld T., Field M.E., Fonarow G.C. (2018). 2017 AHA/ACC/HRS Guideline for Management of Patients with Ventricular Arrhythmias and the Prevention of Sudden Cardiac Death. Circulation.

[22] Kleindorfer D.O., Towfighi A., Chaturvedi S., Cockroft K.M., Gutierrez J., Lombardi-Hill D., Kamel H., Kernan W.N., Kittner S.J., Leira E.C. (2021). 2021 Guideline for the Prevention of Stroke in Patients with Stroke and Transient Ischemic Attack: A Guideline From the American Heart Association/American Stroke Association. Stroke.

[23] Rassi A., Marin-Neto J.A. (2010). Chagas Disease. Lancet.

[24] Aras R., da Matta J.A.M., Mota G., Gomes I., Melo A. (2003). Cerebral Infarction in Autopsies of Chagasic Patients with Heart Failure. Arq. Bras. Cardiol..

[25] Cerqueira-Silva T., Gonçalves B.M., Pereira C.B., Porto L.M., Marques M.E., Santos L.S., Oliveira M.A., Félix I.F., de Sousa P.R.P., Muiños P.J. (2022). Chagas Disease Is an Independent Predictor of Stroke and Death in a Cohort of Heart Failure Patients. Int. J. Stroke.

[26] Oliveira-Filho J. (2009). Stroke and Brain Atrophy in Chronic Chagas Disease Patients: A New Theory Proposition. Dement. Neuropsychol..

[27] Vásconez-González J., Miño C., Izquierdo-Condoy J.S., Salazar-Santoliva C., López-Cortés A., Ortiz-Prado E. (2024). Cardioembolic Stroke in Chagas Disease: Unraveling the Underexplored Connection through a Systematic Review. Trop. Dis. Travel Med. Vaccines.

[28] Guedes P.M.M., de Andrade C.M., Nunes D.F., de Sena Pereira N., Queiroga T.B.D., Machado-Coelho G.L.L., Nascimento M.S.L., Do-Valle-Matta M.A., da Câmara A.C.J., Chiari E. (2016). Inflammation Enhances the Risks of Stroke and Death in Chronic Chagas Disease Patients. PLoS Neglected Trop. Dis..

[29] Vinajá D., Aché A. (2012). Alteraciones Electrocardiográficas en pacientes con Enfermedad de Chagas. Hospital José Rangel de Villa de Cura. 1998–2008. Rev. Inst. Nac. Hig. Rafael Rangel.

[30] Gula L.J., Redfearn D.P., Jenkyn K.B., Allen B., Skanes A.C., Leong-Sit P., Shariff S.Z. (2018). Elevated Incidence of Atrial Fibrillation and Stroke in Patients with Atrial Flutter-A Population-Based Study. Can. J. Cardiol..

[31] Mitchell B. Atrial Flutter—Cardiovascular Disorders. https://www.msdmanuals.com/professional/cardiovascular-disorders/specific-cardiac-arrhythmias/atrial-flutter.

[32] Rassi A., Marin J.A., Rassi A. (2017). Chronic Chagas Cardiomyopathy: A Review of the Main Pathogenic Mechanisms and the Efficacy of Aetiological Treatment Following the BENznidazole Evaluation for Interrupting Trypanosomiasis (BENEFIT) Trial. Mem. Inst. Oswaldo Cruz.

[33] Dias J.C.P., Ramos A.N., Gontijo E.D., Luquetti A., Shikanai-Yasuda M.A., Coura J.R., Torres R.M., Melo J.R.d.C., Almeida E.A.d., Oliveira W.d. (2016). II Consenso Brasileiro em Doença de Chagas, 2015. Epidemiol. E Serviços Saúde.

[34] Hasslocher-Moreno A.M., Saraiva R.M., Sangenis L.H.C., Xavier S.S., de Sousa A.S., Costa A.R., de Holanda M.T., Veloso H.H., Mendes F.S.N.S., Costa F.A.C. (2021). Benznidazole Decreases the Risk of Chronic Chagas Disease Progression and Cardiovascular Events: A Long-Term Follow up Study. EClinicalMedicine.

[35] Gascón J., Albajar P., Cañas E., Flores M., Gómez i Prat J., Herrera R.N., Lafuente C.A., Luciardi H.L., Moncayo Á., Molina L. (2008). Diagnóstico, manejo y tratamiento de la cardiopatía chagásica crónica en áreas donde la infección por Trypanosoma cruzi no es endémica. Enfermedades Infecc. Microbiol. Clínica.

[36] Sousa A.S.d., Xavier S.S., Freitas G.R.d, Hasslocher-Moreno A. (2008). Prevention Strategies of Cardioembolic Ischemic Stroke in Chagas’ Disease. Arq. Bras. Cardiol..

[37] Monteiro J.M.C., San-Martin D.L., Silva B.C.G., Jesus P.A.P.d., Oliveira Filho J. (2018). Anticoagulation in Patients with Cardiac Manifestations of Chagas Disease and Cardioembolic Ischemic Stroke. Arq. Neuropsiquiatr..

[38] Carod-Artal F.J. (2007). Stroke: A Neglected Complication of American Trypanosomiasis (Chagas’ Disease). Trans. R. Soc. Trop. Med. Hyg..

